# Feeling Better Before, Not After: An Ecological Momentary Assessment of Affect Around Exercise in Women With Eating Disorders

**DOI:** 10.1002/eat.70138

**Published:** 2026-06-25

**Authors:** Danielle A. N. Chapa, Kelsie T. Forbush, Yiyang Chen, Courtney E. Costain, Samiya I. Rasheed

**Affiliations:** ^1^ University of Pittsburgh Pittsburgh Pennsylvania USA; ^2^ University of Kansas Lawrence Kansas USA; ^3^ Nova Southeastern University Davie Florida USA

**Keywords:** accelerometry, affect, eating disorders, ecological momentary assessment, maladaptive exercise

## Abstract

**Objective:**

Maladaptive exercise includes excessive, compulsive, or compensatory exercise and is a common eating‐disorder (ED) symptom associated with increased severity, slower rates‐of‐recovery, and faster rates‐of‐relapse. Affect‐regulation theories posit that maladaptive exercise functions to reduce high negative affect (NA), although support for this model is mixed. Previous studies have not integrated ecological momentary assessment (EMA) with accelerometry or examined the affect‐regulation model in individuals with EDs who frequently engage in maladaptive exercise. The objective of this study was to examine trajectories of NA and positive affect (PA) through a 7‐day EMA study combined with accelerometry in women with EDs (*N* = 84).

**Method:**

Piecewise generalized mixed‐effects regression models evaluated the trajectories of PA and NA in the hours leading up to and following self‐reported exercise and exercise identified via accelerometry.

**Results:**

NA was increasing before self‐reported exercise, although NA did not meaningfully change relative to objectively measured exercise. PA was increasing prior to exercise and decreasing after exercise, and this pattern was consistent for both self‐reported and objectively measured exercise. Rates of rising PA were steeper in the hours leading up to higher intensity exercise episodes.

**Discussion:**

Though inconsistent with affect‐regulation models, the current study offers preliminary evidence that exercise is associated with disrupted affective responses among women with EDs who regularly engage in maladaptive exercise. Results suggest that the hours prior to high‐intensity exercise are associated with increasing PA, perhaps due to planning or anticipating exercise, for a considerable proportion of people with EDs. If replicated, treatments may consider decreasing the reward value placed on intense exercise and increasing value placed on low‐intensity or non‐exercise activities.

## Summary

- Maladaptive exercise is a common eating‐disorder behavior that is believed to help individuals with eating disorders regulate their emotions.
- Results found that positive emotions increased before exercise, especially in the hours before intense exercise, and positive emotions decreased after exercise.
- Negative emotions decreased before self‐reported exercise, although there was no change in negative emotions after exercise.
- Results suggested that the hours prior to intense exercise are associated with increasing positive affect, perhaps because planning activity or anticipating exercise expectancies may be rewarding for women with eating disorders.
- If supported by future research, treatments for eating disorders may consider decreasing the reward value placed on high‐intensity exercise and increasing the value placed on non‐exercise activities to increase positive emotions.

## 1 Introduction

Exercise, defined as any physical activity undertaken to improve health or fitness, is associated with benefits to both physical and mental health (Chekroud et al. 2018; Reiner et al. 2013; Rosenbaum et al. 2014). People with eating disorders (EDs) often report engaging in exercise to improve health, for enjoyment, or as a coping strategy for difficult emotions (Lampe et al. 2021). When incorporated appropriately into ED treatment (Cook et al. 2016), exercise can promote improvements in physical health (e.g., increased bone density through strength training) and mental well‐being (Bratland‐Sanda et al. 2012; Hall et al. 2016; Pacanowski et al. 2017). Although exercise may offer benefits for individuals with EDs, it can also become maladaptive when motivated by weight or shape control, used to compensate for eating, or performed in a compulsive manner (Dittmer et al. 2018; Holland et al. 2014; Meyer and Taranis 2011; Noetel et al. 2016). Maladaptive exercise is a broad term meant to capture the full spectrum of harmful exercise. As such, maladaptive exercise may include exercise completed at extreme intensities or durations (i.e., excessive exercise), intended to compensate for loss‐of‐control (LOC) eating (i.e., compensatory exercise), or completed despite serious illness or injury or with extreme rigidity (i.e., compulsive exercise; Adkins and Keel 2005; Dalle Grave et al. 2008; Holland et al. 2014; Mond et al. 2004; Mond et al. 2006; Monell et al. 2018).

Maladaptive exercise is common among individuals with EDs and frequently necessitates intervention due to its link with poor treatment prognosis. Maladaptive exercise is a transdiagnostic ED symptom, present in approximately 80% of individuals with anorexia nervosa (AN), 73% with atypical AN, and 40% with bulimia nervosa (BN) (Gorrell et al. 2021). Individuals who regularly engage in maladaptive exercise report lower quality‐of‐life and higher levels of depression, anxiety, obsessive thoughts, and compulsive behaviors (Peñas‐Lledó et al. 2002; Shroff et al. 2006; Young et al. 2018). Furthermore, maladaptive exercise interferes with treatment and recovery, being associated with greater ED severity, lower remission rates, longer hospitalizations, and faster relapse (Dalle Grave et al. 2008; Monell et al. 2018; Solenberger 2001; Stiles‐Shields et al. 2015; Strober et al. 1997). Treatments targeting maladaptive exercise are critical for improving outcomes and supporting long‐term recovery, although the mechanisms underlying maladaptive exercise remain insufficiently understood.

One promising direction for understanding the mechanisms that maintain maladaptive exercise involves examining the role of affect (Meyer and Taranis 2011), given that (1) exercise is known to modulate affect in the general population (Kanning and Schlicht 2010; Liao et al. 2017), and (2) affect‐regulation models of ED behaviors, such as binge eating and purging, have empirical support (Haedt‐Matt and Keel 2015; Schaefer et al. 2020; Wonderlich et al. 2022). The affect‐regulation model of maladaptive exercise posits that increasing negative affect (NA) triggers maladaptive exercise, which functions to reduce NA. Ecological momentary assessment (EMA) study designs are well suited to identify trajectories of affect leading up to and following a behavior of interest in a naturalistic environment. Two prior studies examined trajectories of affect before and after exercise among individuals with EDs (Engel et al. 2013; Lampe et al. 2023). Contrary to the affect‐regulation model, elevations in positive affect (PA), not NA, preceded exercise (Engel et al. 2013; Lampe et al. 2023), and this trend was observed for both maladaptive and adaptive episodes of exercise. PA increased in the hours following adaptive exercise; however, PA decreased in the hours following maladaptive exercise (Lampe et al. 2023). Together, the existing literature suggests that exercise may be less of a reactive behavior to rising NA. Instead exercise may be more likely to occur when PA is increasing or exercise may represent a highly anticipated planned activity that is associated with increasing PA in the hours prior to the activity. The effect of exercise on PA appears to vary depending on whether the activity is adaptive or maladaptive. Maladaptive exercise may be associated with decreasing PA due to the compulsivity/rigidity of the exercise which may manifest in unhelpful cognitions about exercise, physical pain/discomfort resulting from excessive exercise or exercise despite illness or injury, or social consequences (e.g., missing social commitments). If maladaptive exercise does not serve an affect‐regulation function, there may be other important maintaining mechanisms to consider.

Two key limitations exist in the current understanding of affective antecedents and consequences of maladaptive exercise. *First*, individuals who do and do not report maladaptive exercise in the months preceding naturalistic observation have often been combined resulting in heterogeneous samples. Evidence from non‐ED samples found that pre‐existing tendencies toward maladaptive exercise moderated affect change, such that individuals with higher maladaptive exercise at baseline showed steeper increases in PA prior to exercise relative to individuals with lower levels of maladaptive exercise (Harris et al. 2025). These findings suggest that affective responses surrounding exercise may be different for individuals with established and routine maladaptive exercise tendencies, perhaps due to changes in exercise expectancies and exercise quality (i.e., more rigid or compulsive) following repeated experiences with maladaptive exercise *Second*, prior studies have rarely integrated accelerometer data into EMA designs. The use of accelerometers could enhance precision in identifying exercise onset and offset. Trying to approximate time of exercise in EMA surveys is limited because it relies on participants to initiate an event‐contingent survey, accurately report exercise time retrospectively, or provides only a range of time that exercise occurred (i.e., between two surveys). Incorporation of accelerometers into EMA studies would also allow examination of how exercise dose, the product of exercise intensity and duration (Reed and Ones 2006), influence acute affective change following exercise. This is particularly relevant given prior evidence that moderate doses of exercise (e.g., 30 min of moderate activity) increased PA (Hansen et al. 2001), whereas vigorous or prolonged exercise is associated with decreased PA (Ekkekakis et al. 2011; Reed and Ones 2006). Understanding how dose of exercise (i.e., duration and intensity) shapes affective trajectories could have important clinical implications for tailoring ED treatment approaches in individuals with a historic pattern of maladaptive exercise.

The overall objective of this study was to replicate prior research on the affect‐regulation model of maladaptive exercise using an EMA design, while extending previous work by sampling only women with EDs who engage in regular maladaptive exercise and by combining self‐reported exercise with wrist‐worn accelerometry to examine exercise duration and calories burned (estimated from exercise intensity and duration) as moderators of affective trajectories. We aimed to characterize trajectories of NA and PA leading up to and following: (1) self‐reported and (2) accelerometer‐based exercise. A priori hypotheses were made when this study was first conceptualized in 2018. Consistent with the affect‐regulation model, we hypothesized that increasing NA would precede exercise and affect would improve following exercise (i.e., increasing PA and decreasing NA). We also hypothesized that exercise duration and calories burned would moderate post‐exercise affect trajectories such that exercise that was longer in duration and expended larger amounts of calories would be associated with decreases in post‐exercise PA in line with research in non‐ED samples demonstrating that moderate, but not high doses, of exercise are associated with increased PA following exercise (Hansen et al. 2001; Reed and Ones 2006).

## 2 Method

### 2.1 Participants

Participants (*N* = 84) were recruited from an active registry of community members with EDs and through study flyers posted around the community and on social media (see Figure 1 for recruitment details). Interested participants completed an online screening questionnaire (details available in Supporting Information). Study inclusion criteria were: (1) current *DSM‐5‐TR* ED; (2) age of 18–65 years; (3) female sex; (4) fluency in written and spoken English; and (5) access to a mobile smartphone. Finally, to be included, participants needed to report frequent maladaptive exercise‐ defined as scoring one standard deviation higher than ED outpatients (Coniglio et al. 2018) on a measure of maladaptive exercise, the Eating Pathology Symptoms Inventory (EPSI) Excessive Exercise scale (Forbush et al. 2013), or reporting maladaptive exercise three times per week, on average, in the past 3 months on an adapted version of the Eating Disorder Diagnostic Scale for DSM‐5 that assessed compulsive and compensatory exercise episodes (additional details in Supporting Information). Study exclusion criteria were: (1) medical conditions that affected appetite or body weight (e.g., untreated thyroid disorder, diabetes, current pregnancy); (2) medications that affected appetite or body weight (e.g., corticosteroids, stimulants); (3) current psychiatric diagnosis that could interfere with EMA protocol adherence (e.g., neurological disorder, current psychosis, and/or current manic episode); (4) significant visual impairment that would interfere with viewing and completing EMA surveys; (5) chronic or acute health problems that limited physical activity (e.g., broken leg, sprained ankle, pulled muscles, cardiopulmonary conditions); and (6) Division 1 collegiate athlete status due to high levels of sport‐related activity that may have differential effects on affective trajectories.

**FIGURE 1:**
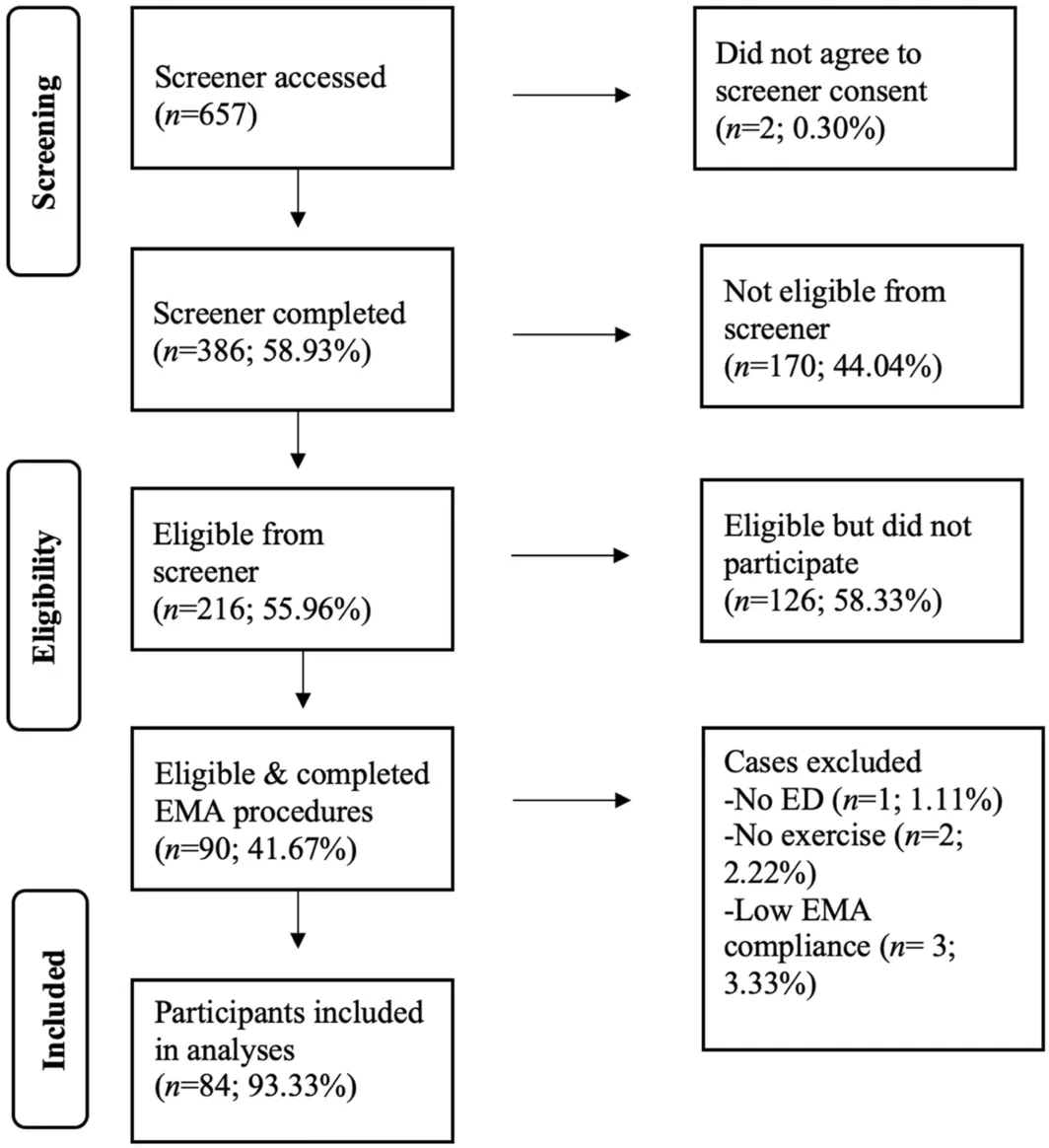
Study recruitment.

### 2.2 Procedure

The University of Kansas Institutional Review Board approved study procedures. Baseline appointments were scheduled for 90 min, either in‐person or virtually. Participants completed two semi‐structured interviews to confirm ED diagnosis and maladaptive exercise behaviors (see Supporting Information for details) and completed self‐report surveys to ascertain demographic data (e.g., age, sex, race, ethnicity). Participants downloaded the PiLR Health EMA App on their personal smartphone and completed a brief training on EMA study procedures (e.g., instructions on using the accelerometer and responding to surveys). Data were collected from 2018 to 2021.

During the 7‐day EMA protocol, participants were instructed to: (1) wear a research‐grade accelerometer, the CentrePoint Insight Watch, on their non‐dominant wrist 24‐h per day; (2) respond daily to six, signal‐contingent surveys on momentary PA and NA; and (3) complete event‐contingent assessments of PA and NA before and after exercise. Participants were instructed to complete event‐contingent surveys any time they considered their behaviors to be exercise (i.e., no specific definition of exercise was provided) to capture any episodes that were conceptualized as exercise by the participant. Participants were signaled six times per day at semi‐random intervals, approximately every 2–3 h, to complete a survey on momentary affect and eating behaviors. Signaled surveys expired after 30 min, and reminder notifications were sent 15 min prior to the expiration. A 7‐day EMA protocol was selected as we expected sufficient sampling of exercise events in 1 week to yield adequate power (see Supporting Information), and 7‐day EMA designs have been used previously to understand eating‐disorder behaviors (Forester et al. 2023; Romano et al. 2025). To limit reactivity effects and any adverse effects associated with self‐monitoring, participants did not have access to their physical activity data on the study watch screen (see Supporting Information for additional EMA details). Participants earned $2.00 each day they wore the accelerometer for at least 12‐waking hours. If participants completed 34 or more assessments of affect, either signal‐ or event‐contingent, they received an additional $36.00 for maximum compensation of $50.00.

### 2.3 EMA Measures

#### 2.3.1 Positive and Negative Affect Schedule (PANAS)

The PANAS (Watson et al. 1988) is comprised of 20 items that assess general PA and NA. To limit participant burden and increase EMA adherence, an adapted, shorter version of the PANAS was used in the current study with permission from the author (D. Watson, personal communication, 2/15/2019). The original PANAS was reduced from 20 items to 10 items for the current study (see Supporting Information for details). Selected PA items included: interested, enthusiastic, determined, strong, and inspired. Selected NA items included: scared, nervous, distressed, ashamed, and irritable. The adapted version of the PANAS was administered to assess momentary PA and NA. The PANAS demonstrated evidence for excellent internal consistency when measuring momentary affect (Watson et al. 1988) (PA *ω* = 0.91 and NA *ω* = 0.82 in the current study). The PANAS has shown evidence for excellent convergent validity with other measures of PA and NA (Watson et al. 1988). The PA and NA subscales of the PANAS demonstrated discriminant validity from one another and compared to measures of anxiety, depression, and general psychological distress (Watson and Clark 1984; Watson et al. 1988; Tellegen 1985). The within‐person correlation between PA and NA was 0.31 providing evidence that while related, PA and NA represented distinct constructs in the current study.

#### 2.3.2 CentrePoint Insight Watch (CP Insight)

The CP Insight Watch is a research‐grade, wrist‐worn accelerometer manufactured by Ametris (previously ActiGraph). During the 7‐day EMA study period, participants were instructed to wear the CP Insight watch on the wrist of their non‐dominant hand for 24 h per day, except when their wrist would be submerged underwater for extended periods. The CP Insight Watches were added to the protocol after the first 10 participants completed the study, and two participants' data were not available due to technical errors when initializing the device. CP Insight Watches recorded raw acceleration data using a triaxial accelerometer that quantifies movement in three different planes or axes, and raw data were summarized into activity counts (i.e., value indicating the intensity of movement/physical activity). The CP Insight watches were configured following standardized procedures, processed to identify time of moderate‐to‐vigorous physical activity (MVPA), and used to estimate calories burned (see Supporting Information). Previous research demonstrated acceptable reliability of the CP Insight watch across placements (dominant vs. non‐dominant wrist) in free‐living conditions (Lee et al. 2025) consistent with prior research evaluating wrist placement of other wrist‐worn triaxial accelerometers (Buchan et al. 2019; Dieu et al. 2017). Non‐dominant wrist placement was selected in the current study as it is associated with a more conservative estimate of MVPA and step counts compared to placement on the dominant wrist (Lee et al. 2025).

### 2.4 Statistical Approach

This study aimed to characterize trajectories of NA and PA leading up to and following: (1) self‐reported and (2) accelerometer‐based exercise. Self‐reported exercise was identified as the time that an exercise event‐contingent survey was submitted. When self‐reported exercise occurred more than once in a day, only the first exercise event was used and only the affect ratings before the second event were modeled to avoid conflating two different exercise events (i.e., the affect ratings following the first exercise event were modeled only as the post‐exercise trajectory of the first event but not also entered as the pre‐exercise trajectory of the subsequent event). Accelerometer‐based exercise time was identified as the start time of the longest MVPA episode derived from the CP Insight watch (see data processing details in Supporting Information). Moderate and vigorous intensities of exercise were combined to ensure adequate power due to a low likelihood that there would be sufficient sampling of purely moderate and purely vigorous intensities to compare differences. Thus, self‐reported exercise time represented events that participants conceptualized as exercise whereas accelerometer‐based exercise time was operationalized using researcher‐imposed definitions (i.e., longest bout of MVPA).

Multilevel modeling (MLM) techniques were implemented in R Version 4.4.1. The lme() function within the nlme package (Pinheiro et al. 2025) was used to build a piecewise generalized mixed‐effects regression model consistent with recommendations for testing affect‐regulation models in EDs (Berg et al. 2013). Momentary affective observations (level 1) were nested within individual participants (level 2). Within‐person residuals were modeled with a continuous autoregressive correlation structure to account for the impact of time and unequal distances between assessment time points. Time of exercise was mean centered at zero, and piecewise linear and quadratic functions of standardized time relative to exercise (i.e., hours prior to exercise and hours following exercise) predicted log transformed PA and NA. Log transformations were completed due to positive skewness of PA and NA ratings. Following best practices for building MLM models to analyze EMA data (Schwartz and Stone 1998), a series of nested models were constructed incrementally adding polynomial linear and quadratic terms, among which we selected the best‐fitting model based on model comparison results. See Table 1 for nested model details. Slopes of the best‐fitting models were used for hypothesis testing. *T*‐tests for the pre‐exercise trajectories test if slope estimates are significantly different from zero. *T*‐tests for the post‐exercise trajectories test if slope estimates are significantly different from the pre‐exercise trajectory representing a significant change in direction or acceleration of affect. Additional analyses were completed to test if the post‐exercise slope estimates were significantly different from zero by fitting a different model with alternative parameterization. Finally, MVPA duration and calories burned were entered as continuous moderators of objectively measured exercise to examine the extent that they impacted the slope of affect in the hours prior to and following exercise. See Supporting Information for additional analytic details, R code, and R output. Data may be available upon request.

**TABLE 1:** Fixed and random effects tested in nested models.

Model	Fixed effects	Random effects
Model A		Random ID Intercept
Model B	(exerciseHours) (exerciseHours:postExercise)	Random ID Intercept
Model C	(exerciseHours) (exerciseHours:postExercise) (exerciseHours)^2^ (exerciseHours:postExercise)^2^	Random ID Intercept
Model D	(exerciseHours) (exerciseHours:postExercise) (exerciseHours)^2^ (exerciseHours:postExercise)^2^ (exerciseHours)^3^ (exerciseHours:postExercise)^3^	Random ID Intercept

*Note:* An “exerciseHours” variable was created to represent the number of standardized decimal hours; negative values represented the number of hours prior to exercise, and positive values represented the number of hours after exercise. A “postExercise” dummy coded variable was created; “0” values were assigned to affect ratings made in the hours prior to exercise, and “1” values were assigned to affect ratings made in the hours after exercise. The “exerciseHours” and “exerciseHours:postExercise” effects were entered as predictors to model the pre‐exercise and post‐exercise trajectories of affect respectively. Squared values represent quadratic effects. After the best fitting model selected with fixed effects, additional random effects (beyond ID) were added sequentially in the following order: exerciseHours, exerciseHours:postexercise, exerciseHours2, exerciseHours2:postexercise, and so forth. Fixed effects represent the average slope of positive and negative affect in the hours prior to and following exercise across the full sample, whereas random effects account for individual variation in those slopes. All models in the current study accounted for individual variation in fixed effects by modeling ID as a random effect.

## 3 Results

A total of *N* = 90 female participants were consented into the study and attended the baseline appointment. There were *n* = 3 participants who were not eligible following the baseline interviews and were excluded from the study. An additional *n* = 3 participants were excluded from analyses due to low EMA compliance (i.e., less than 50%). Thus, the final sample used for analyses was *N* = 84. The mean (SD) age was 29.83 (11.49) years with a range of 18–64 years, and the mean BMI was 25.41 (*5.57*) with a range of 17.64–39.85. Most participants had a diagnosis of “Other Specified Feeding or Eating Disorder” (69.05%; *n* = 58) followed by BN (27.38%; *n* = 23), AN (2.38% *n* = 2), and binge eating disorder (BED) (1.19%, *n* = 1). Most participants were White, Non‐Hispanic, and college‐educated. Approximately one‐third of the sample reported currently receiving mental‐health treatment. Of the 28 receiving current treatment, the majority reported receiving treatment for anxiety or a phobia (*n* = 21) followed by depression (*n* = 19), obsessive compulsive disorder (*n* = 5), substance use (*n* = 1), and “other” (*n* = 7). Other reasons for seeking mental health treatment included an eating disorder (*n* = 2), attention deficit hyperactivity disorder (*n* = 1), grief from loss (*n* = 1), postpartum experiences (*n* = 1), anxiety/stress management (*n* = 1), and panic attacks (*n* = 1) (Table 2).

**TABLE 2:** Self‐reported participant demographics and eating‐disorder history.

Variable	*n* (%/SD)
Race/ethnicity	
Non‐Hispanic	71 (84.52%)
White	69 (82.14%)
Asian	4 (4.76%)
Multi‐racial	2 (2.38%)
Another race	2 (2.38%)
Missing race	7 (8.33%)
Mental health	
Lifetime eating disorder treatment	10 (11.90%)
Current mental health treatment	28 (33.33%)
Current psychiatric medication use	22 (26.19%)
Education/employment	
Less than high school diploma or GED	1 (1.19%)
High school diploma or GED	5 (5.95%)
Some college	20 (23.81%)
Associate's degree	3 (3.57%)
Bachelor's degree	22 (26.19%)
Master's degree	19 (22.62%)
Doctorate degree	6 (7.14%)
Employed	62 (73.81%)

Body mass index	*M* (SD)
Measured body mass index	25.56 (5.47)
Self‐reported body mass index	24.41 (5.57)

*Note:* Self‐report data is from 77 participants. Seven participants did not return their self‐report packets. Percentages were based on the full sample of 84 participants. Prior to COVID‐19 restrictions, height and weight were measured in‐person at baseline using a wall‐mounted stadiometer and scale. When in‐person research appointments were no longer possible, participants were asked to sign a release of information for their medical provider to verify their most recent height and weight measurements. When height and weight could not be verified by a medical provider in the past 3 months, self‐reported height and weight was used. The correlation between measured BMI and self‐reported BMI was 0.86, providing support for using self‐reported height and weight when weight verification was not possible.

Participants completed an average of 34.4 signal‐contingent surveys per week and an average of 7.15 event‐contingent surveys per week corresponding to 275 self‐reported exercise events. Participants were highly compliant with signal‐contingent surveys (mean = 81.92%, median = 85.71%) with a range of 14–42 surveys. Of the 72 individuals with accelerometer data available, participants had, on average, 6.5 days of valid accelerometer data (i.e., wear times of at least 12 waking hours) and 208 accelerometer‐identified exercise events. Across days with both self‐reported and accelerometer‐based exercise times (*N* = 148), there was a moderate amount of overlap (*r* = 0.58) between 24‐h decimal clock times across methods.

### 3.1 Self‐Reported Exercise Results

Detailed model comparison results are available in the Supporting Information. Model C, with two fixed linear and two fixed quadratic slopes, emerged as the best fitting model for PA and NA. The addition of random effects for the pre‐ and post‐ exercise slopes did not improve model fit. PA increased curvilinearly in the hours leading up to exercise (linear estimate = 0.013, quadratic estimate = 0.077; Table 3) indicating that the rate of increase in PA accelerated in the hours closest to exercise (Figure 2). Following exercise, the linear slope of PA significantly deflected down (linear deflection = −0.309; Table 3) and PA decreased curvilinearly in the hours following exercise (linear estimate = −0.182, SE = 0.029, *t* = −6.241, *p* < 0.001; quadratic estimate = 0.051, SE = 0.020, *t* = 2.501, *p* = 0.013). In summary, PA increased in the hours prior to self‐reported exercise and decreased in the hours following self‐reported exercise.

**TABLE 3:** Multilevel models for positive and negative affective relative to self‐reported exercise time.

	Positive affect			Negative affect		
	Est.	SE	*t*	Est.	SE	*t*
Intercept	2.496	0.036	69.677***	1.931	0.032	60.523***
Hours prior to event	0.127	0.016	8.098***	−0.078	0.014	−5.535***
(Hours prior to event)^2^	0.077	0.017	4.647***	−0.059	0.015	−3.953***
Hours following event	−0.309	0.041	−7.467***	0.121	0.037	3.273**
(Hours following event)^2^	−0.027	0.019	−1.426	0.041	0.017	2.416*

*Note:* Est. = log‐link estimates; SE = standard error. Fixed slope estimates for the pre‐exercise and post‐exercise trajectories are provided for the 275 self‐reported exercise episodes. Pre‐exercise and post‐exercise trajectories were centered on exercise time defined as the submission of an exercise event‐contingent survey. The pre‐exercise linear and quadratic slope estimates are represented by Hours prior to event and (Hours prior to event)^2^, respectively. The post‐exercise linear and quadratic slope estimates are represented by Hours following event and (Hours following event)^2^, respectively. Significant *t*‐tests for the pre‐exercise trajectories suggest that the slope estimates are significantly different from zero. Significant *t*‐tests for the post‐exercise trajectories suggest that the slope estimates are significantly different from the pre‐exercise trajectory. Additional analyses were completed to test if the post‐exercise trajectory slopes were significantly different from zero. Results from those analyses found that the positive affect post‐exercise trajectory linear and quadratic slopes were significantly different from zero. The negative affect linear and quadratic slope estimates for the post‐exercise trajectory were both not significantly different from zero.

* *p* < 0.05.

** *p* < 0.01.

*** *p* < 0.001.

**FIGURE 2:**
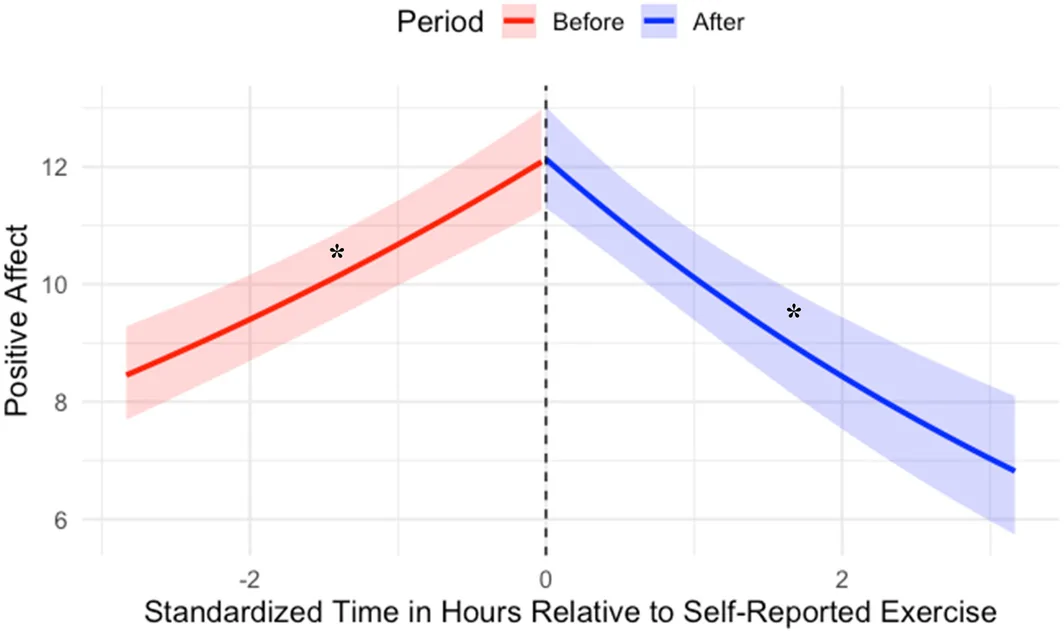
Positive affect trajectories pre‐ and post‐ self‐reported exercise. *Note:* Trajectories of positive affect are shown in the hours leading up to exercise (pre‐exercise trajectory) and in the hours following exercise (post‐exercise trajectory). The pre‐ and post‐exercise trajectories are centered on zero, and zero represents the time that self‐reported exercise occurred. This plot was created after back‐transforming the previously logged transformed positive and negative affect values to aid in interpretation of the data. In the 3 h leading up to exercise, PA scores increased 4 points, on average. In the 3 h after exercise, PA decreased 5 points, on average. *The slope of the trajectory is significantly different from zero.

NA decreased curvilinearly in the hours leading up to exercise (linear estimate = −0.078, quadratic estimate = −0.059; Table 3) indicating that the rate of decrease in NA accelerated in the hours closest to exercise (Figure 3). Following exercise, the linear and quadratic slopes of NA deflected upward (linear deflection = 0.121, quadratic estimate = 0.041; Table 3). NA stabilized following exercise (i.e., slope not significantly different from zero; linear estimate = 0.043, SE = 0.026, *t* = 1.668, *p* = 0.095; quadratic estimate = −0.018, SE = 0.018, *t* = −0.993, *p* = 0.321). In summary, NA declined in the hours prior to self‐reported exercise and became constant after self‐reported exercise.

**FIGURE 3:**
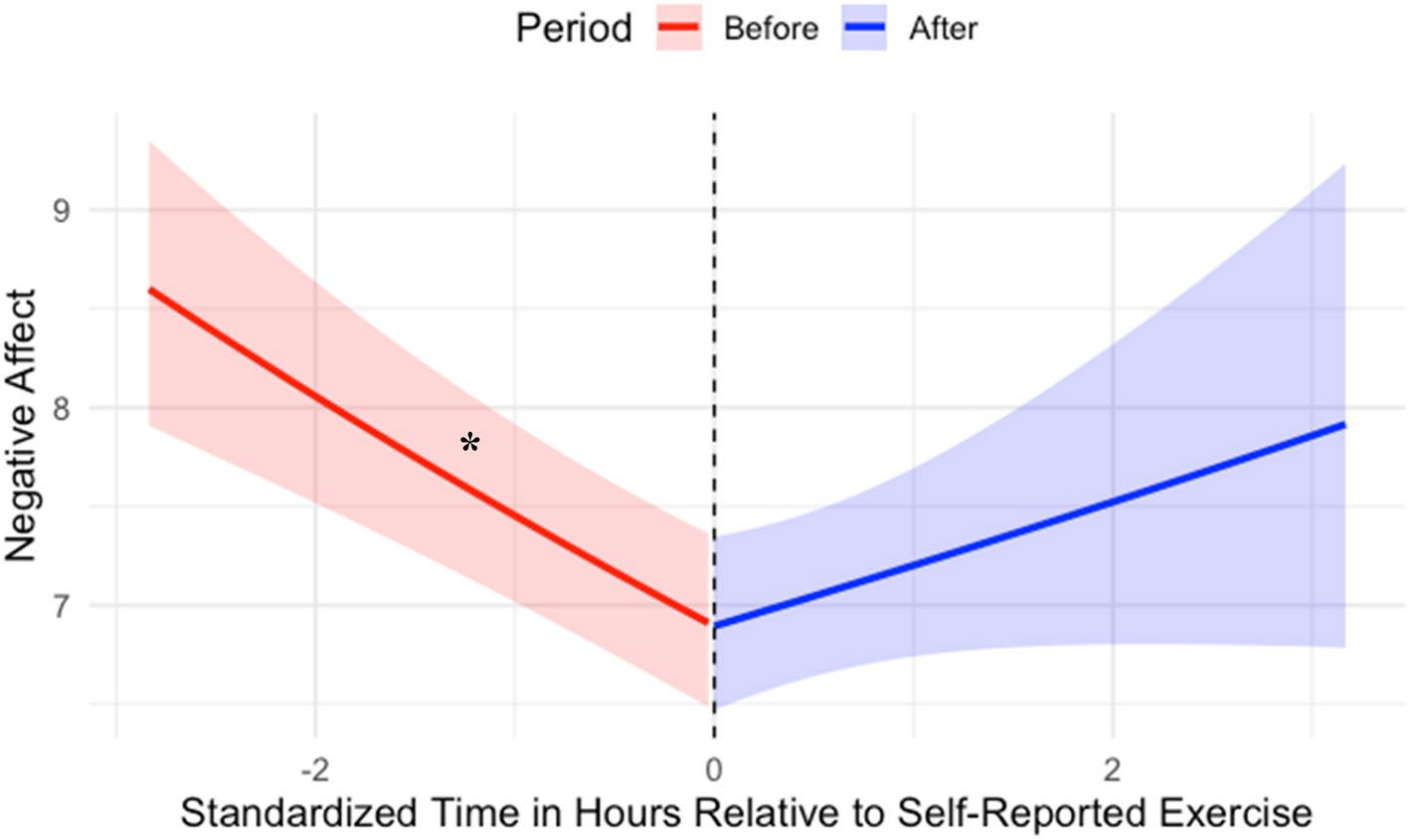
Negative affect trajectories pre‐ and post‐ self‐reported exercise. *Note:* Trajectories of negative affect are shown in the hours leading up to exercise (pre‐exercise trajectory) and in the hours following exercise (post‐exercise trajectory). The pre‐ and post‐exercise trajectories are centered on zero, and zero represents the time that self‐reported exercise occurred. This plot was created after back‐transforming the previously logged transformed positive and negative affect values to aid in interpretation of the data. In the 3 h leading up to exercise, NA decreased 2 points. *The slope of the trajectory is significantly different from zero.

### 3.2 Objectively Measured Exercise Results

Detailed model comparison results are available in the Supporting Information. Model C, with two fixed linear and two fixed quadratic slopes, emerged as the best fitting fixed effects model for PA. Model A, with no fixed slopes and only a random intercept, emerged as the best fitting model for NA. PA increased curvilinearly in the hours leading up to exercise (linear estimate = 0.134, quadratic estimate = 0.111; Table 4). Thus, the rate of increase in PA accelerated in the hours closest to exercise (Figure 4). Following exercise, the trajectory of PA significantly deflected downward (linear deflection = −0.348; Table 4) and began decreasing (linear estimate = −0.214, SE = 0.039, *t* = −5.422, *p* < 0.001; quadratic estimate = 0.075, SE = 0.025, *t* = 3.060, *p* = 0.002). In summary, PA increased in the hours prior to MVPA and decreased in the hours following MVPA. The NA slopes were not estimated because the best fitting model included only a random intercept indicating there was no significant change in NA prior to or following exercise. Caloric expenditure emerged as a significant moderator of pre‐exercise change in PA, such that PA increased at a steeper rate in the hours prior to exercise that had higher caloric expenditure.

**TABLE 4:** Multilevel models for positive and negative affective relative to objectively measured exercise time.

	Positive affect			Negative affect		
	Est.	SE	*t*	Est.	SE	*t*
Intercept	2.504	0.047	52.883***	2.026	0.036	56.071***
Hours prior to event	0.134	0.025	5.329***	—	—	—
(Hours prior to event)^2^	0.111	0.024	4.608***	—	—	—
Hours following event	−0.348	0.061	−5.696***	—	—	—
(Hours following event)^2^	−0.035	0.022	−1.595	—	—	—

*Note:* Est. = log‐link estimates; SE = standard error. Fixed slope estimates for the pre‐exercise and post‐exercise trajectories are provided for the 208 accelerometer‐identified episodes. Pre‐exercise and post‐exercise trajectories were centered on accelerometer‐based physical activity time defined as the longest bout of moderate‐to‐vigorous activity. The pre‐exercise linear and quadratic slope estimates are represented by Hours prior to event and (Hours prior to event)^2^. The post‐exercise linear and quadratic slope estimates are represented by Hours following event and (Hours following event)^2^. Significant *t*‐tests for the pre‐exercise trajectories suggest that the slope estimates are significantly different from zero. Significant *t*‐tests for the post‐exercise trajectories suggest that the slope estimates are significantly different from the pre‐exercise trajectory. Thus, in this table, the post‐exercise trajectory estimates represent the amount of change or deflection in slope that was observed following exercise. Additional analyses were completed to test if the post‐exercise trajectory slopes were significantly different from zero. Results from those analyses found that the positive affect post‐exercise trajectory linear and quadratic slopes were significantly different from zero.

*** *p* < 0.001.

**FIGURE 4:**
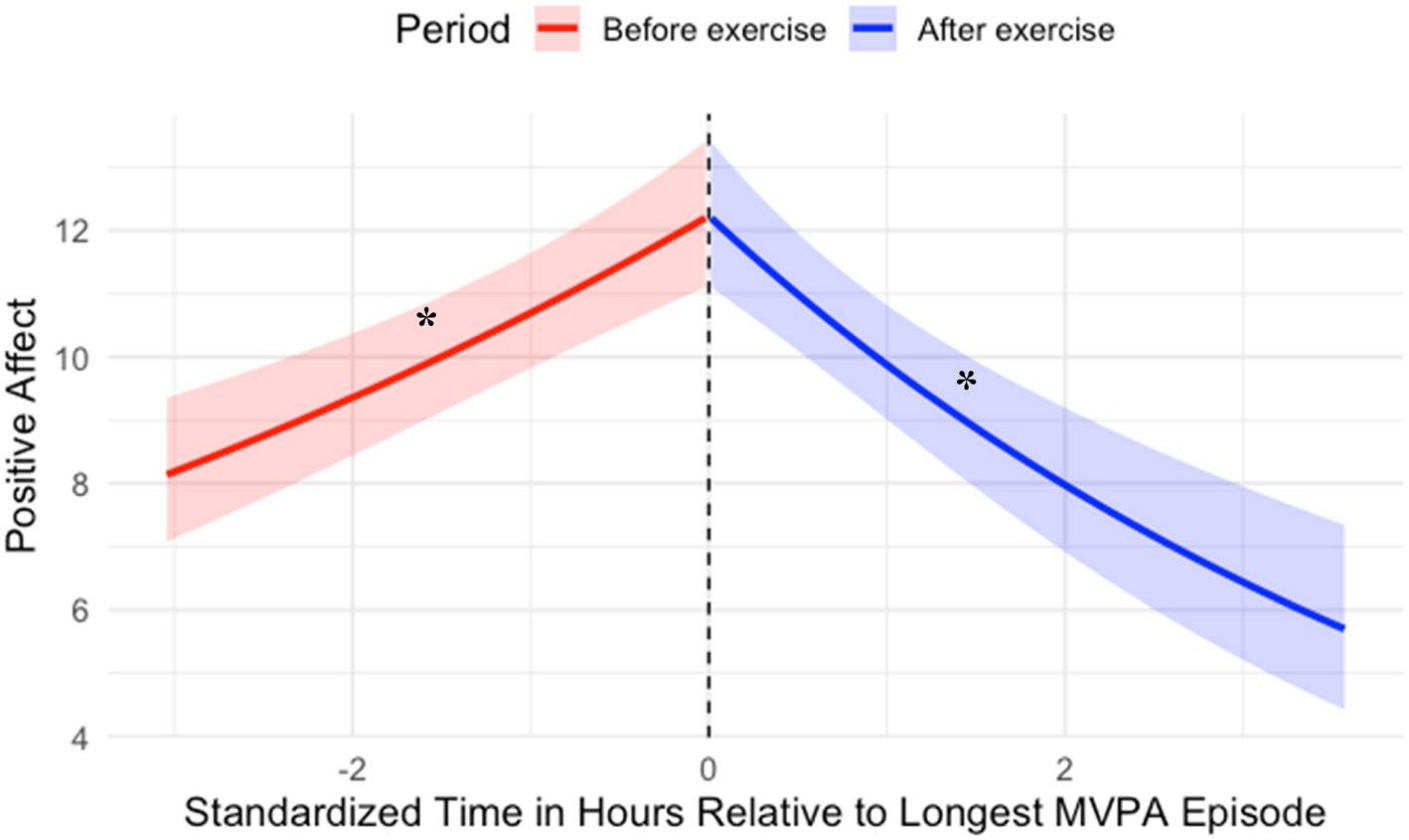
Positive affect trajectories pre‐ and post‐objectively measured exercise. *Note:* MVPA = moderate‐to‐vigorous physical activity. Trajectories of positive affect are shown in the hours leading up to exercise (pre‐exercise trajectory) and in the hours following exercise (post‐exercise trajectory). The pre‐ and post‐exercise trajectories are centered on zero, and zero represents the time that objectively measured exercise (i.e., longest bout of moderate‐to‐vigorous physical activity) occurred. This plot was creating after back‐transforming the previously logged transformed positive and negative affect values to aid in interpretation of the data. In the 3 h leading up to exercise, PA scores increased 4 points, on average. In the 3 h after exercise, PA decreased 5 points, on average. *The slope of the trajectory is significantly different from zero.

## 4 Discussion

The objective of this study was to examine trajectories of PA and NA leading up to and following exercise using both self‐reported and accelerometer‐based exercise. We hypothesized that increasing NA would precede exercise and decreasing NA would follow exercise consistent with the affect‐regulation model. Contrary to our hypotheses, NA did not decrease following exercise. Rather, feelings of NA decreased in the hours leading up to self‐reported exercise. This finding did not replicate when examining NA change prior to objectively identified MVPA. NA did not change in the hours leading up to or following objectively identified MVPA. These differences may have emerged due to differences in exercise‐intent. All self‐reported exercise episodes were internally conceptualized as “exercise” in order for participants to initiate an event‐contingent survey, whereas accelerometer‐based findings were derived from a mix of physical activity episodes that may or may not have been conceptualized as “exercise.” Future EMA designs could consider leveraging research‐grade accelerometers to prompt individuals to self‐report their exercise‐intent when physical activity is detected. It is also possible that differences in NA in the hours leading up to exercise were observed because self‐reported exercise always captured the first exercise episode, regardless of duration or intensity, as opposed to the accelerometer‐based approach which captured the longest episode of MVPA. Overall, the affect‐regulation model of maladaptive exercise was not supported, highlighting that maladaptive exercise may have a different function in individuals with an ED. Although increasing NA did not precede exercise at the hourly level, it is possible the affect‐regulation function of exercise operates on a delayed time scale such that NA increases intent to exercise at a later time though actual exercise may not be feasible until days or hours later.

PA increased and NA decreased in the hours prior to self‐reported exercise. Prior EMA studies also observed increases in PA occurring before exercise (Engel et al. 2013; Lampe et al. 2023). One possibility is that improving affect led to an increased likelihood of spontaneous (i.e., non‐planned) exercise or physical activity. Given that the majority of participants reported planning their days around exercise at baseline and that many end‐of‐day signal‐contingent surveys included a response from participants that they were indeed planning exercise for the following day (see Supporting Information), it appears that anticipating or planning exercise may be one factor contributing to improved affect. A risk and maintenance model of BN proposed that some ED behaviors transition from being impulsive during the early stages of an ED to more habitual as the ED progresses (Pearson et al. 2015). This theory could help contextualize the unexpected pre‐exercise trajectory of increasing PA and decreasing NA observed in the current study. Many individuals with EDs engage in exercise to reduce NA (Bratland‐Sanda et al. 2010; Noetel et al. 2016); perhaps exercise serves a negative reinforcement function via reductions in NA in early stages. Over time, NA may decline prior to exercise due to the anticipation/expectation that NA will decrease when exercise occurs based on learned associations. Exercise expectancies were not assessed in the current study, and future research is needed to test whether the relationships between maladaptive exercise and momentary affect are mediated by expectations of affect change as a result of future exercise and plans to exercise. For example, is higher within‐person NA concurrently or prospectively associated with higher exercise expectancies, higher exercise intentions, and a higher likelihood of planning later exercise?

Another unexpected result was that PA decreased following exercise. Although not hypothesized, this finding is consistent with some prior EMA research (Engel et al. 2013; Lampe et al. 2023). For example, Lampe et al. (2023) found that maladaptive exercise was associated with decreasing PA and adaptive exercise was associated with increasing PA. In the current study, decreases in PA were not moderated by MVPA duration or calories burned highlighting that the decline in PA following exercise was not a function of exercise duration or estimated caloric expenditure. It is possible that decreased PA following exercise is related to distorted cognitions about exercise performance or the extent to which exercise goals were achieved (e.g., distorted evaluations about whether a workout was “good” or “bad” based on number of calories burned). Body checking or self‐comparison during or immediately following exercise could similarly influence changes in affect (e.g., upward social comparisons during exercise).

Several limitations of the current study are important to discuss. First, the current study was limited to a sample of primarily White women and, therefore, the experiences of men and diverse races and ethnicities were not represented in the current study. Second, data collection of the current study overlapped with the COVID‐19 pandemic. Thus, our results could have been influenced by the ongoing stressors and business closures associated with navigating the pandemic. For example, there was a period when gyms and other exercise facilities were closed. Qualitative and quantitative research on the effects of COVID‐19 on EDs found that individuals reported exercising more overall, exercising more at home and outdoors, exercising more intensely, and feeling a higher drive to exercise (Brown et al. 2021; Schlegl et al. 2020; Termorshuizen et al. 2020). These studies suggest that maladaptive exercise likely became more frequent during the COVID‐19 pandemic. Third, individual exercise episodes during the EMA period where not distinguished as maladaptive versus adaptive. Similarly, specific forms of maladaptive exercise (e.g., compensatory vs. compulsive) were not disentangled from one another. The current study was designed to establish affective trajectories surrounding exercise among women with EDs who frequently engaged in maladaptive exercise. A portion of exercise episodes in the current study (identified by either self‐report or accelerometer) could have been more adaptive in nature, and different forms have maladaptive exercise could have been associated with distinct affective trajectories. Future EMA studies should consider collecting fine‐grained information about specific exercise episodes to determine the extent that they were adaptive versus maladaptive, and if maladaptive, the extent that they were compensatory and/or compulsive in nature. Fourth, although the study was strengthened by objective physical activity data, some important qualities of exercise were missing, including objective and subjective assessments of exertion (e.g., heart rate, V02max, self‐reported perceived exertion), which could be important moderators of post‐exercise affect. Future research focused on estimating proximal affect changes surrounding exercise in free‐living environments (as opposed to lab settings) may consider incorporating accelerometers with heart rate capabilities and a rating of self‐perceived exertion to better approximate exercise dose, above‐and‐beyond MVPA duration and calories burned estimated from movement. Finally, potential limitations of the EMA design include possibility of reactivity effects associated with responding to prompts about mood and wearing an accelerometer. Many individuals with EDs already track their eating and physical activity using apps and passive sensors, which may limit the amount of reactivity effects associated with the EMA protocol. Additionally, some design features of the study were intended to limit reactivity effects, including one practice day and disenabling the function on the study accelerometer that displays exercise metrics to participants. Although the study accelerometer display was locked to limit reactivity, participants were not precluded from wearing their own device in addition to the study device during the course of the study.

Despite limitations, the current study had several strengths. EMA procedures allowed for a longitudinal assessment of real‐time changes in PA and NA relative to exercise. Physical activity and reports of affect were measured in free‐living environments rather than in a laboratory or hospital setting, increasing ecological validity. Exercise time was evaluated using both self‐reported initiation of exercise and MVPA identified with wrist‐worn accelerometry. Self‐reported exercise and wrist‐worn accelerometry are associated with their own limitations and strengths. Self‐reported exercise episodes require that participants remember to log an exercise episode in a timely manner, although one major strength is that it captures self‐perceived exercise episodes regardless of duration or intensity of physical activity. The identification of exercise using an accelerometer is limited to the researcher‐imposed definitions of what constitutes as an exercise episode. In the current study, our standardized definition of an MVPA episode could have excluded some types of exercise (e.g., traditional strength training with longer breaks or lower‐intensity physical activity), although the addition of the accelerometer allowed us to assess the extent that MVPA duration and calories burned moderate affective trajectories. Finally, the current study also included a sample of individuals with a wide range of BMIs.

Although replication of the study results is needed, our results may have important implications for the treatment of maladaptive exercise among individuals with EDs. Given that PA increased in the moments before exercise and decreased in the moments after exercise, treatment that focuses on helping people with EDs increase PA through the identification of enjoyable activities, other than exercise, could be helpful. Thus, scheduling positive activities using Behavioral Activation (Martell et al. 2021) could be beneficial for reducing maladaptive exercise. Positive Affect Treatment (PAT), which is an adapted version of Behavioral Activation, was recently proposed to treat difficulties in reward processing among individuals with AN (Haynos et al. 2021) and may be efficacious for improving maladaptive exercise as well. Results highlight that women with EDs who engage in regular maladaptive exercise may not be receiving the affective benefits of exercise as demonstrated by decreased PA following exercise. Treatments aimed at helping individuals with EDs exercise in more adaptive ways should consider strategies to help people exercise in ways that improve their affect.

In conclusion, the current study used an EMA design combined with accelerometry to better understand exercise behaviors relative to momentary changes in affect. Preliminary longitudinal evidence suggested that exercise is associated with disrupted affective responses among women with EDs who regularly engage in maladaptive exercise. Results consistently demonstrated that PA improved in the hours leading up to exercise, especially in the hours before intense exercise, as evidenced by increasing PA. In the hours after exercise, PA demonstrated a decreasing trajectory raising questions as to what interferes with the mood‐boosting components of exercise for this population. Results suggested that the hours prior to intense exercise are associated with increasing positive affect, perhaps because planning activity or anticipating exercise expectancies, may be rewarding for women with EDs. If replicated, treatments may consider decreasing the reward value placed on high‐intensity exercise and increasing the value placed on non‐exercise activities to increase PA.

## Author Contributions

**Yiyang Chen:** formal analysis, writing – review and editing. **Danielle A. N. Chapa:** conceptualization, data curation, formal analysis, funding acquisition, investigation, methodology, project administration, writing – original draft. **Samiya I. Rasheed:** writing – review and editing. **Kelsie T. Forbush:** conceptualization, resources, supervision, writing – review and editing. **Courtney E. Costain:** writing – review and editing.

## Funding

Research reported in this publication was supported by the National Institute of Mental Health of the National Institutes of Health under award number R36MH120943. DANC's time was partially supported by the National Institute of Mental Health under award number T32MH018269. The content is solely the responsibility of the authors and does not necessarily represent the official views of the National Institutes of Health.

## Disclosure

Use of AI was not involved in any component of preparation of this manuscript.

## Lived Experience Involvement Statement

No specific efforts were undertaken to involve persons with lived experience in the study design or execution, or in the preparation of this manuscript.

## Conflicts of Interest

The authors declare no conflicts of interest.

## Supporting information


**Table S1:** Frequency of baseline excessive exercise behaviors.
**Table S2:** Positive affect and negative affect model building comparisons for self‐reported exercise time‐fixed effects only.
**Table S3:** Positive affect and negative affect model building comparisons for objectively measured exercise time‐fixed effects only.

## Data Availability

R code for statistical analyses is available within the Supporting Information. Additional study materials (e.g., study protocol, data) may be available upon request.
